# Baicalein Triggers Mitochondria-Mediated Apoptosis and Enhances the Antileukemic Effect of Vincristine in Childhood Acute Lymphoblastic Leukemia CCRF-CEM Cells

**DOI:** 10.1155/2013/124747

**Published:** 2013-01-28

**Authors:** Yun-Ju Chen, Chieh-Shan Wu, Jeng-Jer Shieh, Jyh-Horng Wu, Hsing-Yu Chen, Ting-Wen Chung, Yu-Kuo Chen, Chi-Chen Lin

**Affiliations:** ^1^Department of Child Care, College of Humanities and Social Sciences, Southern Taiwan University of Science and Technology, Tainan 71005, Taiwan; ^2^Department of Dermatology, Kaohsiung Veterans General Hospital, Kaohsiung, Taiwan; ^3^Department of Dermatology, Faculty of Medicine, College of Medicine, Kaohsiung Medical University, Kaohsiung, Taiwan; ^4^Graduate Institute of Medicine, College of Medicine, Kaohsiung Medical University, Kaohsiung, Taiwan; ^5^Institute of Biomedical Sciences, College of Life Science, National Chung Hsing University, Taichung, Taiwan; ^6^Department of Medical Research and Education, Taichung Veterans General Hospital, Taichung, Taiwan; ^7^Department of Forestry, National Chung Hsing University, Taichung, Taiwan; ^8^Department of Food Science, National Pingtung University of Science and Technology, Pingtung, Taiwan

## Abstract

Acute lymphoblastic leukemia (ALL) accounts for approximately 75% of childhood leukemia, and chemotherapy remains the mainstay therapy. Baicalein is an active flavonoid used in traditional Chinese medicine and has recently been found to have anticancer, anti-inflammatory, and antiallergic properties. This study aims to investigate the molecular apoptotic mechanisms of baicalein in CCRF-CEM leukemic cells and to evaluate the combined therapeutic efficacy of baicalein with several commonly used chemotherapeutic drugs in CCRF-CEM cells. Our results demonstrate that baicalein induces mitochondria-dependent cleavage of caspases-9 and -3 and PARP with concomitant decreases in IAP family proteins, survivin, and XIAP. Furthermore, our results present for the first time that baicalein triggers a convergence of the intrinsic and extrinsic apoptotic pathways via the death receptor-caspase 8-tBid signaling cascade in CCRF-CEM cells. In addition, we also present for the first time that the combination of baicalein and vincristine results in a synergistic therapeutic efficacy. Overall, this combination strategy is recommended for future clinical trials in the treatment of pediatric leukemia owing to baicalein's beneficial effects in alleviating the vomiting, nausea, and skin rashes caused by chemotherapy.

## 1. Introduction

Leukemia is often described as acute or chronic. Acute refers to the relatively short time course of the disease (being lethal in as little as a few weeks if left untreated) to distinguish it from the very different disease of chronic lymphocytic leukemia, which has a potential time course of many years. Almost all childhood leukemia is acute. Acute lymphocytic (lymphoblastic) leukemia (ALL) accounts for approximately 75% of childhood leukemia. The treatment of childhood ALL consists of a combination of several anticancer drugs and is usually divided into the following 3 phases: induction, consolidation (also called intensification) and maintenance [1]. Vincristine and methotrexate are two drugs commonly used to treat childhood ALL that can be used in all 3 phases of treatment [1]. Depending on the therapy dose, most chemotherapeutic agents have side effects, such as diarrhea, nausea, vomiting, and skin rashes. For example, vincristine has additional peripheral neurological side effects such as hearing changes, sensory loss, numbness, and tingling. Serious side effects in response to chemotherapeutic agents prompt researchers and clinicians to seek novel anticancer agents that have fewer side effects, and these newly explored anticancer agents can be used in combination with the commonly used chemotherapeutic agents to reduce serious side effects.

 Baicalein, extracted from the root of *Scutellaria baicalensis*, is an active flavonoid that has been found to have anticancer properties [2–4]. Baicalein inhibits cancer cell proliferation and induces apoptosis of human gastric cancer cell lines [5]. In an *in vivo* experiment, mice were injected with bladder cancer cells with concurrent oral administration of a high-baicalein-yielding supplement in one group or with no baicalein supplementation in the control group. All the control mice had a progressive increase in the tumor volume over the ensuing days of the study, whereas the mice treated with baicalein (Scutellaria) had significant inhibition of the tumor growth [6]. Other studies testing baicalein as an antitumor agent support its potential use in breast and prostate cancers [7–9]. Baicalein has been found to selectively induce apoptosis in human cancer cell lines with minimal influence on noncancer cells [10–12]. In fact, *Scutellaria baicalensis* has been used in traditional Chinese medicine to treat a variety of medical conditions including diarrhea, vomiting, nausea, asthma, gout, and inflammatory conditions, such as dermatitis, arthritis, bronchitis, and inflammatory bowel disease [2]. Although baicalein is found to induce apoptosis in several types of cancers, the molecular apoptotic mechanisms of baicalein and the combined effects of baicalein with other commonly used chemotherapeutic drugs on childhood acute lymphoblastic leukemia CCRF-CEM cells have not previously been investigated.

In the present study, we aimed to investigate the molecular apoptotic effects and mechanisms of baicalein on CCRF-CEM leukemic cells and evaluate the combined therapeutic efficacy of baicalein with other commonly used chemotherapeutic drugs on CCRF-CEM leukemic cells. We found that baicalein induces apoptosis primarily through the mitochondria-dependent activation of the caspase-9 and -3 pathways. Moreover, we demonstrated for the first time that baicalein induces the activation of the death receptor-caspase 8-tBid signaling cascade, which converges with the intrinsic pathway at the mitochondrial level. More importantly, we found a synergistic therapeutic effect when baicalein and vincristine were used in combination.

## 2. Material and Methods

### 2.1. Chemicals and Reagents

Baicalein (Sigma-Aldrich, St. Louis, MO) was 98% pure. A 100 mM stock solution of baicalein was prepared, and this solution was subsequently diluted to 6.25–100 *μ*M in DMSO. Vincristine (Sigma-Aldrich, St. Louis, MO) was >95% pure. A 100-*μ*M stock solution of Vincristine was prepared, and this solution was subsequently diluted to 1.25–5 nM in DMSO. MTT, 3-(4,5-dimethylthiazoly1-2)-2,5-diphenyltetrazoliumbromide, Dantrolene dimethyl sulfoxide (DMSO), propidium iodide (PI), and Triton X-100 were purchased from Sigma. RPMI Media 1640, fetal bovine serum (FBS), and trypsin-EDTA were obtained from Gibco. The JC-1 Assay was obtained from Molecular Probes. Inhibitors of caspase-3 (Ac-DEVD-CMK), caspase-8 (Z-IETD-FMK), and caspase-9 (Z-LEHD-FMK) were obtained from Calbiochem and were dissolved in DMSO.

### 2.2. Cell Culture

The human childhood leukemia cell line (CCRF-CEM) was purchased from ATCC. The cells were grown in RPMI 1640 medium (GIBCO) with 20% fetal bovine serum (FBS; GIBCO) at 37°C in a humidified incubator containing 5% CO_2_.

### 2.3. Antibodies

Antibodies against Bcl-2 and Bax were purchased from Santa Cruz. An antibody against Cytochrome c was purchased from BD PharMingen. Antibodies specific for cleaved caspases-3, -8, and -9, truncated Bid, XIAP, cleaved PARP, and Survivin were purchased from Cell Signaling. An anti-*β*-actin antibody was purchased from Sigma. Anti-mouse and -rabbit IgG peroxidase-conjugated secondary antibodies were purchased from Jackson ImmunoResearch Laboratory. The DR4, DR5, Fas, TRAIL, FasL antibodies used in flow cytometry were obtained from eBioscience.

### 2.4. Cytotoxicity Assay

The cytotoxic effect of baicalein on CCRF-CEM cells was measured by the 3-[4,5-dimethylthiazol-2-yl]-2,5 diphenyltetrazolium (MTT) assay (Sigma-Aldrich, St. Louis, MO). CCRF-CEM cells were seeded onto 24-well plates for 24 h. Various concentrations of baicalein were added to the cells, followed by 24 h and 48 h of incubation. Then, the medium was removed, and 200 *μ*L of 1x MTT solution was added to each well for 4 h. The absorbance at 540 nm was determined via a microplate reader (TECAN). The half maximal inhibitory concentration (IC 50) of the drug was calculated by polynomial regression analysis using Microsoft Excel software. 

### 2.5. Annexin V Assay

An apoptosis assay was conducted with the BioVision Annexin V-FITC Apoptosis Detection Kit. Leukemia CCRF-CEM cells were seeded onto a 6-cm dish for 24 h and then exposed to different doses of baicalein for 24 h. Cells were harvested and washed twice with PBS and then resuspended in 500 *μ*L of binding buffer. Cell suspensions were then incubated with 5 *μ*L of annexin V-FITC and 5 *μ*L of propidium iodide (PI) for 10 min at room temperature in the dark. The cells were immediately evaluated by flow cytometry (FACSCalibur, BD BioSciences).

### 2.6. Cell Cycle Analysis

3 × 10^5^ cells were plated onto a 6 cm dish for 24 h. The cells were then treated with different concentrations of baicalein for 24 h, followed by cell collection with centrifugation. The pellets were mixed with 75% ethanol at −20°C overnight. The cells were centrifuged and resuspended in 500 *μ*L of PI staining solution (2 mg/mL RNase, 1 mg/mL PI and 0.5% Triton X-100) for 1 h at 37°C in the dark. The cells were then analyzed by FACS Calibur flow cytometry (BD Biosciences). The distribution and percentages of cells in the sub-G1, G0/G1, S, and G2/M phases of the cell cycle were analyzed using WinMDI software (Scripps Research Institute, La Jolla, CA).

### 2.7. Measurement of the Reactive Oxygen Species (ROS) by Flow Cytometry

ROS production was measured after staining the cells with 5-(and-6)-carboxy-2′,7′-dichlorodihydrofluorescein diacetate (DCFDA; Molecular Probes). Additionally, 3 × 10^5^ CCRF-CEM cells/dish were plated onto a 6-cm tissue culture dish with 3 mL of medium. The medium was removed 12 and 24 h after baicalein treatment, and culture medium containing 5 *μ*M DCFDA was added under low-light conditions. The cells were incubated for 30 min at 37°C, and the ROS level was measured by flow cytometry (FACSCalibur, BD BioSciences).

### 2.8. Western Blot Analysis

Whole cells were lysed in 2% SDS containing 10 mM EDTA, 50 mM Tris base, 10% SDS, pH 8.0, and boiled at 100°C for 10 minutes. Protein concentrations were measured by a BCA Protein Assay. Equal amounts of protein were loaded into 10%–15% SDS-PAGE gels, transferred to PVDF membranes and blocked with 5% non-fat milk in TBST buffer (20 mM Tris-HCl, 120 mM NaCl, and 0.1% Tween 20) for 1 hour. Membranes were incubated with various primary antibodies. After washing, the blots were incubated with HRP-labeled mouse and rabbit secondary antibodies (Jackson Lab) for 2 hours. The signals of the blots were then developed using the enhanced chemiluminescence (ECL) system and analyzed by the LAS3000 system (*Fujifilm*, Tokyo, Japan). Densitometric analysis was performed with ImageJ software (National Institute of Health, Bethesda, MD, USA).

### 2.9. Mitochondrial Membrane Potential Assay

The mitochondria-specific cationic dye JC-1 (Invitrogen, Carlsbad, CA), which undergoes potential-dependent accumulation in the mitochondria, was used. When the membrane potential (ΔΨ) is below 120 mV, JC-1 remains monomeric and emits a green light (540 nm) following excitation by blue light (490 nm). At membrane potentials higher than 120 mV, JC-1 monomers aggregate and emit red light (590 nm) following excitation by green light (540 nm). CCRF-CEM cells were seeded onto a 6-well plate for 24 hr and were then treated with various concentrations of baicalein for 24 h, followed by staining with 25 *μ*M JC-1 for 30 min at 37°C. Cells were then washed in PBS, and 10,000 cells of each sample were analyzed by flow cytometry (FACS Calibur, BD, USA). 

### 2.10. Mitochondria/Cytosol Fractionation

The Mitochondria/Cytosol Fractionation Kit (Biovision) was used for isolation of the mitochondrial fraction from the cytosolic fraction in mammalian cells. Cells were harvested and washed twice with PBS and resuspended in 500 *μ*L of Cytosol extraction buffer mix on ice for 10 minutes. Then, cells were homogenized by Dounce Tissue Grinder (Biovision) on ice for 30–50 passes, followed by centrifugation at 3000 rpm for 10 minutes at 4°C. The supernatants were collected carefully, and centrifugation was repeated again at 13000 rpm for 30 minutes at 4°C. The final supernatants were collected as the cytosolic fractions.

### 2.11. Caspase Inhibitor and the Antagonistic Anti-Fas Antibody ZB-4 Assay

The cells were seeded at 4 × 10^4^ cells/well in 24-well tissue culture plates. After 18 h, the cells were pre-treated with inhibitors of caspase-3 (Ac-DEVD-CMK), caspase-8 (Z-IETD-FMK), and caspase-9 (Z-LEHD-FMK) and an antagonistic anti-Fas antibody ZB-4 (R&D) for 1 hr. Then, the cells were treated with the indicated concentrations of baicalein for 24 hr. Then, the treated cells were incubated with 5 mg/mL MTT for 4 hr at 37°C, followed by removal of the supernatant and developing the color by adding 600 *μ*L of DMSO to each well. The absorbance was read at 570 nm on a microplate reader (Tecan, Durham, NC).

### 2.12. Transient Transfection and RNA Interference

CCRF-CEM cells were grown to 30% to 50% confluency in 6-well plates and transfected with 400 pmol of FADD siRNA (Santa Cruz) with lipofectamine 2000 reagent (Invitrogen). Cells were harvested and reseeded 24 h after transfection, and cell viability was assessed by the MTT assay in the presence or absence of baicalein.

### 2.13. Analysis of Death Receptors and Ligands by Flow Cytometry

Analyses of the death receptor membrane expression and death receptor ligand expression were performed by flow cytometry. 3 × 10^5^ cells/dish were plated onto 6-cm tissue culture dishes in 3 mL of medium, followed by incubation with 0, 25, 50, and 100 *μ*M of baicalein for 24 hr. After trypsinizing and washing cells with PBS, the treated cells were stained with anti-DR4-FITC, anti-DR5-FITC, anti-Fas-FITC, anti-TRAIL-FITC and anti-FasL-FITC (eBioscience) at 4°C for 30–45 minutes. The protein expressions of the treated cells were analyzed by flow cytometry (FACSCalibur, BD BioSciences).

### 2.14. Measurement of the Intracellular Free Calcium Level

Approximately 3 × 10^5^ CCRF-CEM cells/well were seeded onto 6 cm culture dishes overnight. The cells were exposed to the indicated concentrations of baicalein for 24 h. Then, the cells were harvested and washed twice with 1 × PBS containing 1% BSA. The cells were stained with 0.5 mL Fluo-3/AM (2.5 *μ*g/mL) (Sigma-Aldrich) for 30 minutes at room temperature in the dark. The stained cells were immediately analyzed by flow cytometry (FACSCalibur, BD, USA).

### 2.15. Statistical Analysis

The results were expressed as the mean ± SD. Statistical analyses were performed by one-way ANOVA followed by Tukey's post hoc test (GraphPad Software Inc., San Diego, CA). *P* values <0.05 was considered statistically significant. In addition, a two-way ANOVA with Bonferroni's test (GraphPad Software Inc., San Diego, CA) was used to test for an interaction between baicalein and vincristine. Results determined by this method were validated further in CCRF-CEM cell line by an isobologram according to “Drug Synergism: Its Detection and Applications” reported by Tallarida [13].

## 3. Results

### 3.1. Baicalein Induces a Potent Apoptotic Effect on CCRF-CEM Leukemic Cells

**Figure 1:**
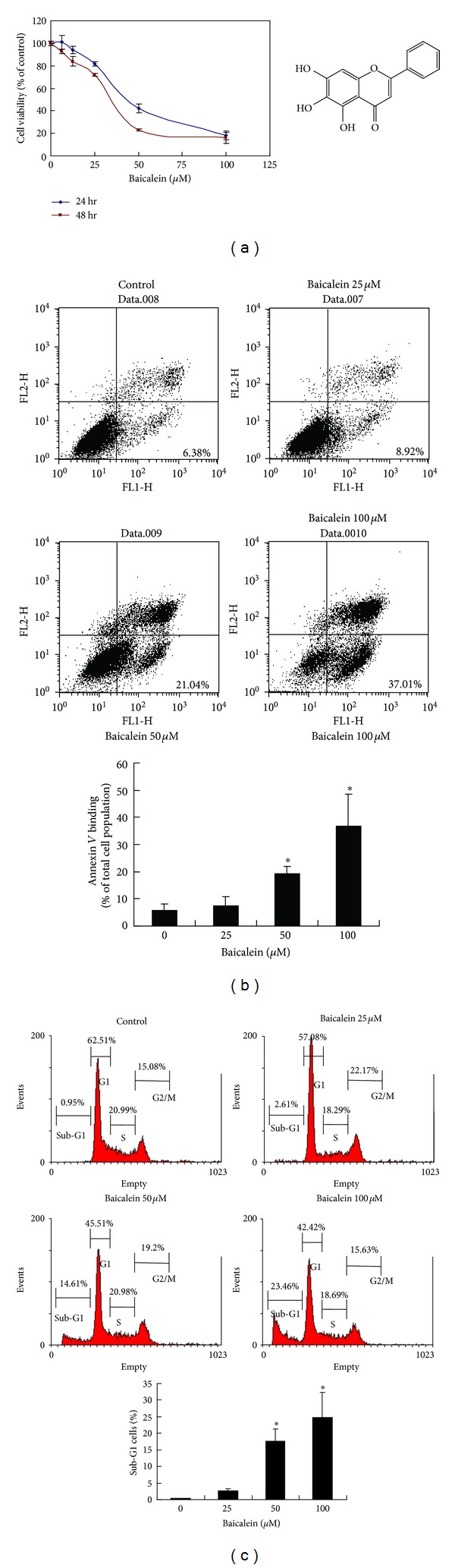
The effects of baicalein on cell viability and apoptosis in CCRF-CEM cells.  (a) 4 × 10^4^ CCRF-CEM cells/well were seeded in 24-well tissue culture plates and were then treated with various concentrations of baicalein for 24 h and 48 h. The cell viability was determined by the MTT assay. Each point on the graph represents the mean ± SD from triplicate wells. Similar results were obtained in three independent experiments. (b) Flow cytometry analysis of baicalein-induced apoptosis in CCRF-CEM cells. The cells were treated with the indicated concentrations of baicalein for 24 h, followed by staining with FITC-Annexin-V and PI. The data are representative of three independent experiments showing similar results. Means ± SD of the experimental replicates is presented with bar graph in the bottom. *P < 0.05, compared with the untreated control group. (c) Cells were treated with the indicated concentrations of baicalein for 24 h, after which the sub-G1 population was determined by propidium iodide (PI) staining and subsequent flow cytometry analysis. The data are representative of three independent experiments showing similar results. Means ± SD of the experimental replicates is presented with bar graph in the bottom. *P < 0.05, compared with the untreated control group.

To determine the effect of baicalein on cell growth, CCRF-CEM leukemic cells were challenged with various doses of baicalein for 24 and 48 hours, and cell viability was measured by the MTT assay. As shown in Figure 1(a), baicalein markedly reduced the cell viability of CCRF-CEM cells in a dose- and time-dependent manner. The IC50 values following 24 and 48 hr of treatment were approximately 48.63 ± 0.78 *μ*M and 33.43 ± 1.948 *μ*M, respectively. To clarify the type of cell death elicited by baicalein, CCRF-CEM cells were treated with various concentrations of baicalein and subjected to flow cytometry analysis following staining with Annexin V-FITC and propidium iodide (PI). As shown in Figure 1(b), the percentages of early apoptotic death (Annexin V+/PI−, lower right quadrant) increased in a dose-dependent manner in CCRF-CEM cells. In addition, the sub-G1 population also increased in a dose-dependent manner 24 hr after baicalein treatment (Figure 1(c)). These results suggest that baicalein induces apoptosis in CCRF-CEM leukemic cells.

### 3.2. Baicalein Triggers Mitochondrial Membrane Potential Loss and Cytochrome c Release

**Figure 2:**
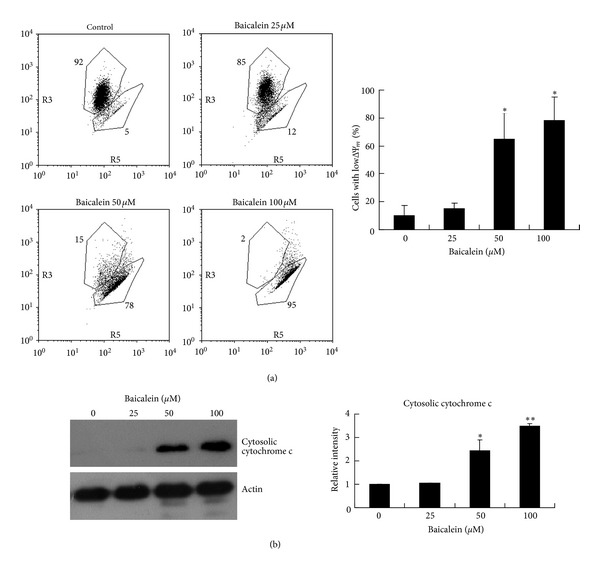
The effects of baicalein on the mitochondrial membrane potential and cytochrome c release in CCRF-CEM cells. (a) The cells were stained with JC-1 fluorescence dye, followed by flow cytometry analysis. The data are representative of three independent experiments with similar results. Means ± SD of the experimental replicates is presented with bar graph in the bottom. *P < 0.05, compared with the untreated control group. (b) The cytosolic fraction of lysates was subjected to SDS-PAGE, followed by Western blot analysis with the anti-cytochrome c antibody. Bands were analyzed by ImageJ and normalized to actin. The data are representative of three independent experiments showing similar results. Means ± SD of the three independent experiments is presented with bar graph in the bottom. *P < 0.05, compared with the untreated control group. **P < 0.01, compared with the untreated control group.

Mitochondria are known to play a central role in apoptotic signaling because the intrinsic and the extrinsic pathways can converge at the mitochondrial level and trigger mitochondrial membrane potential loss [14, 15]. To investigate whether mitochondria are involved in baicalein-induced apoptosis, we used the fluorescent cationic dye JC-1 to examine the effect of baicalein on the mitochondrial membrane potential (Δ*ψ*_*m*_). As illustrated in Figure 2(a), baicalein at 24 hr caused a dose-dependent decrease in the red fluorescence and a concomitant dose-dependent increase in the green fluorescence in CCRF-CEM cells, indicating that baicalein triggers a dose-dependent reduction in Δ*ψ*_*m*_. Cytochrome c release from the mitochondria into the cytosol is an important event for apoptosis induction following loss of Δ*ψ*_*m*_ [16]. Therefore, we examined the cytosolic fraction of CCRF-CEM cells with western blotting after 24 hr of baicalein exposure. As shown in Figure 2(b), baicalein caused a dose-dependent elevation in cytochrome c expression. These results verify that the mitochondria are involved in baicalein-induced apoptosis.

### 3.3. Baicalein Induces Apoptosis Primarily via the Mitochondrial Pathway in CCRF-CEM Leukemic Cells

**Figure 3:**
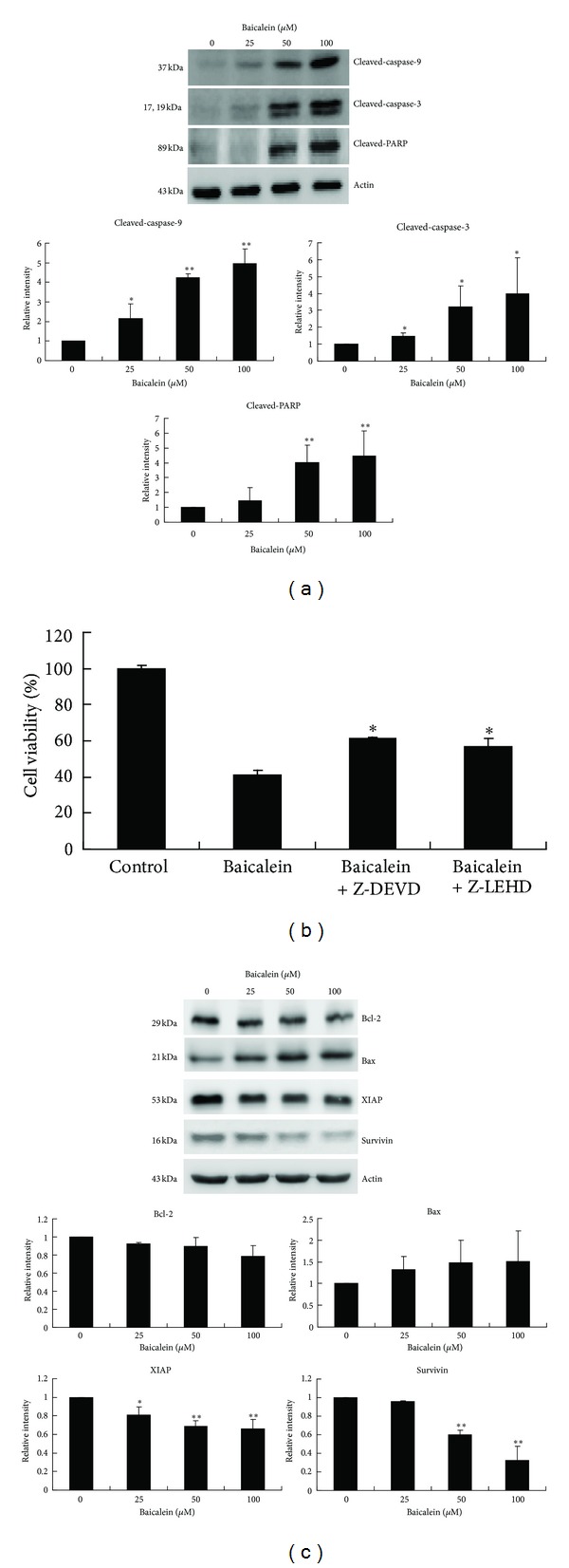
The effects of baicalein on mitochondrial pathway-related protein expression in CCRF-CEM cells. (a) Cells were treated with the indicated concentrations of baicalein for 24 hr, and the cleaved forms of caspase-9, caspase-3, and PARP were determined by immunoblotting. The data are representative of three independent experiments showing similar results. Means ± SD of the three independent experiments are presented with bar graph on the right. *P < 0.05, compared with the untreated control group. (b) Cells pretreated with caspase 3 inhibitor, z-DEVD-fmk, or caspase-9 inhibitor Z-LEHD-fmk were incubated with baicalein for 24 h, after which the cell viability was determined by the MTT assay. The data are expressed as the mean ± SD from triplicate wells. Similar results were obtained in three independent experiments. *P < 0.05, compared with the baicalein only group. (c) Cells were treated with the indicated concentrations of baicalein for 24 hr, and the effects of baicalein on the expression of Bcl-2 family proteins and IAPs were determined by immunoblotting. Similar results were obtained at least twice. For (a) and (c), bands were analyzed by ImageJ and normalized to actin. The data are representative of three independent experiments showing similar results. Means ± SD of the three independent experiments are presented with bar graph on the right. *P < 0.05, compared with the untreated control group. **P < 0.01, compared with the untreated control group.

Mitochondrial dysfunction triggers subsequent activation of caspase-9 in the presence of Apaf-1, which in turn results in the activation of downstream caspase-3 and cleavage of PARP. To investigate the molecular events following mitochondrial dysfunction, apoptotic related proteins were examined with a Western blott. As shown in Figure 3(a), 24 hr of treatment with baicalein induced a dose-dependent increase in the cleaved/activated forms of caspase-9, caspase-3, and PARP in CCRF-CEM cells. Treatment with the caspase-3 inhibitor Z-DEVD and caspase-9 inhibitor Z-LEHD could rescue CCRF-CEM cells from baicalein-induced cell death, indicating that the caspase-dependent pathway was involved (Figure 3(b)). In addition, Bcl-2 family proteins are frequently involved in mitochondria dysfunction [17], and IAP family members can mediate the inhibition of caspases-9 and -3, thereby suppressing apoptosis [18]. As illustrated in Figure 3(c), 24 hr of baicalein treatment resulted in significant reductions of XIAP and Survivin protein expressions. Although there was no significant change in Bcl-2 and Bax expressions, we still observed a slight decrease in Bcl-2 and a slight increase in Bax protein expressions. Taken together, these findings indicate that baicalein induces apoptosis primarily via the intrinsic mitochondrial pathway.

### 3.4. Activation of the Caspase-8-tBid Pathway in Baicalein-Induced Apoptosis

**Figure 4:**
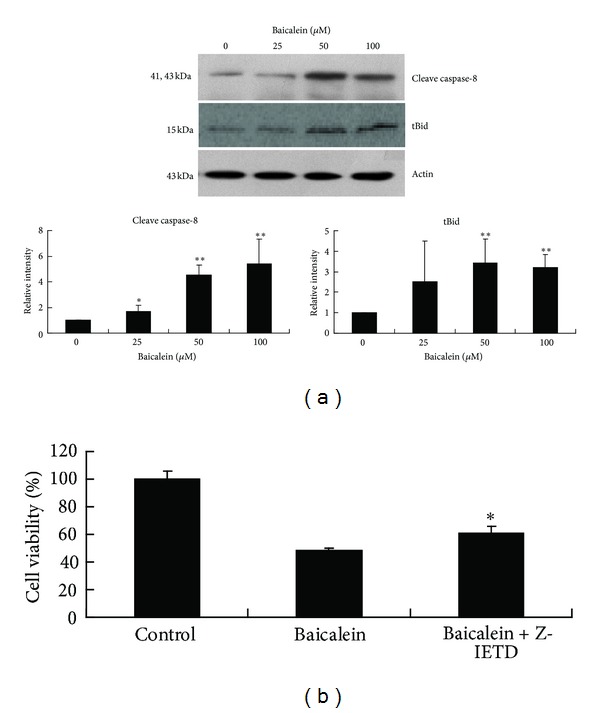
The effects of baicalein on caspase-8 and Bid cleavage. (a) CCRF-CEM cells were treated with the indicated concentrations of baicalein for 24 hr. Western blot analysis was carried out to detect the cleaved forms of caspase 8 and Bid. The data correspond to a representative of three independent experiments with similar results. Bands were analyzed by ImageJ software and normalized to actin. The data are representative of three independent experiments showing similar results. Means ± SD of the three independent experiments is presented with bar graph on the right. *P < 0.05, compared with the untreated control group. **P < 0.01, compared with the untreated control group. (b) Cells pretreated with caspase 8 inhibitor, z-IETD-fmk, were incubated with baicalein for 24 h, after which the cell viability was determined by the MTT assay. The data are expressed as the mean ± SD from triplicate wells. Similar results were obtained in three independent experiments. *P < 0.05, compared with the baicalein only group.

It is well established that caspase-8 can directly activate caspase-3 through cleavage and/or indirectly cleave Bid (23kd) into truncated Bid (tBid), which triggers mitochondria-dependent activation of the caspase-9 and -3 cascade [19]. To determine whether baicalein could induce the activation of caspase 8 and its downstream Bid, CCRF-CEM cells treated with 24 hr of baicalein were subjected to Western blot analysis. As shown in Figure 4(a), the cleaved caspase-8 and tBid protein levels increased after baicalein treatment. In addition, pretreatment with a selective caspase-8 inhibitor, z-IETD-fmk, could partially rescue cell viability (Figure 4(b)). These data indicate that the caspase 8-tBid pathway participates in the mitochondria-mediated apoptosis triggered by baicalein. 

### 3.5. ROS Is Not Involved in Baicalein-Induced Apoptotic Cell Death

**Figure 5:**
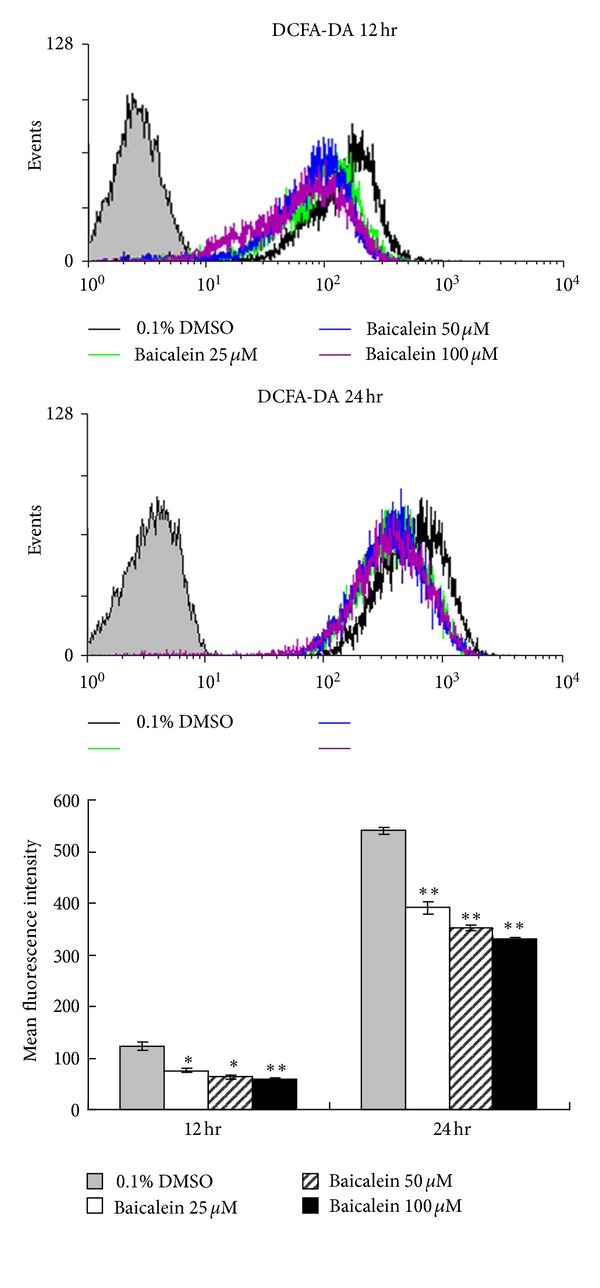
The effect of baicalein on ROS production in CCRF-CEM cells. Cells were stained with DCFDA, followed by flow cytometry analysis 12 h or 24 h post various concentrations of baicalein treatment. The mean fluorescence intensities for ROS generation were tabulated. The data are represented as the mean ± SD in triplicate tests. Similar results were obtained in three independent experiments. *P < 0.05, compared with the untreated control group (0.1% DMSO). **P < 0.01, compared with the untreated control group.

Reactive oxygen species (ROS) are highly reactive molecules that have been implicated in the induction or enhancement of apoptosis [20]. A previous study suggests that ROS can induce apoptosis through the caspase-8/Bid/Bax pathway in human lymphocytes [21]. However, baicalein is known to exhibit antioxidant effects [22, 23]. Therefore, we investigated whether baicalein could induce ROS generation by means of a fluorescent probe, DCFA-DA. As shown in Figure 5, 12 hr and 24 hr of baicalein exposure resulted in a dose-dependent decrease in DCFA-DA fluorescence intensity in CCRF-CEM cells, indicating that ROS did not participate in baicalein-induced apoptosis.

### 3.6. Baicalein Activates the Death Receptor Pathway

**Figure 6:**
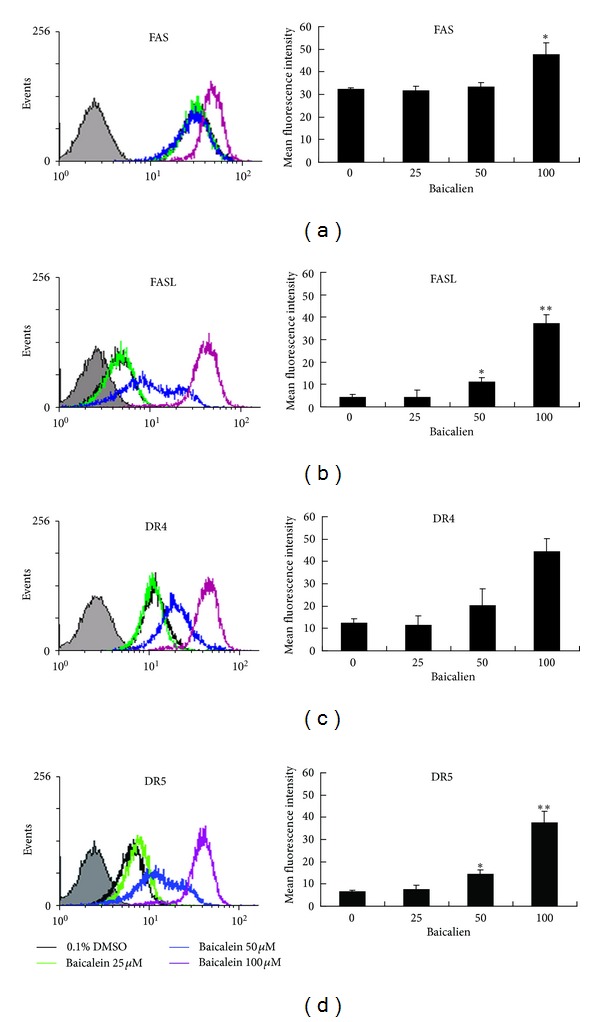
Involvement of the death receptor pathway in baicalein-induced apoptosis. Flow cytometric analysis of (a) Fas, (b) FasL, (c) DR4, and (d) DR5 expression levels following 24 h of various concentrations of baicalein exposure. The mean fluorescence intensities for each death receptor/ligand expression were tabulated. The data are represented as the mean ± SD in duplicate tests. The data are representative of three independent experiments showing similar results. *P < 0.05, compared with the untreated control group. **P < 0.01, compared with the untreated control group.

Our present study observes that baicalein can induce caspase-8 activation. It is well established that caspase-8 activation is associated with death receptor signaling. Six distinct death receptors are known, including the TNF receptor-1 (TNF-R1), Fas (APO-1/CD95), TRAMP or death receptor-3 (DR3), TRAIL receptor-1 and receptor-2 (TRAIL-R1/DR4, TRAIL-R2/DR5), and death receptor-6 (DR6). Among them, the TRAIL and Fas receptors and their ligands have been extensively investigated [24]. To determine whether death receptors were involved in baicalein-induced caspase-8 activation and apoptosis, the surface expression level of these death receptors was determined by flow cytometry following 24 hr of various doses of baicalein treatment. As illustrated in Figures 6(a) and 6(b), the Fas receptor increased significantly only after 100 *μ*M of baicalein treatment, whereas FasL increased significantly at 50 *μ*M and elevated largely at 100 *μ*M. Figures 6(c) and 6(d) showed that DR4 and DR5 increase significantly at 50 *μ*M and increased greatly at 100 *μ*M. Overall, these results demonstrate that the death receptors are involved in baicalein-induced caspase-8 activation and apoptosis.

### 3.7. The Caspase-8 Activation-Dependent Death Receptor Pathway Is Not the Only Trigger for the Mitochondria-Mediated Apoptosis Induced by Baicalein

**Figure 7:**
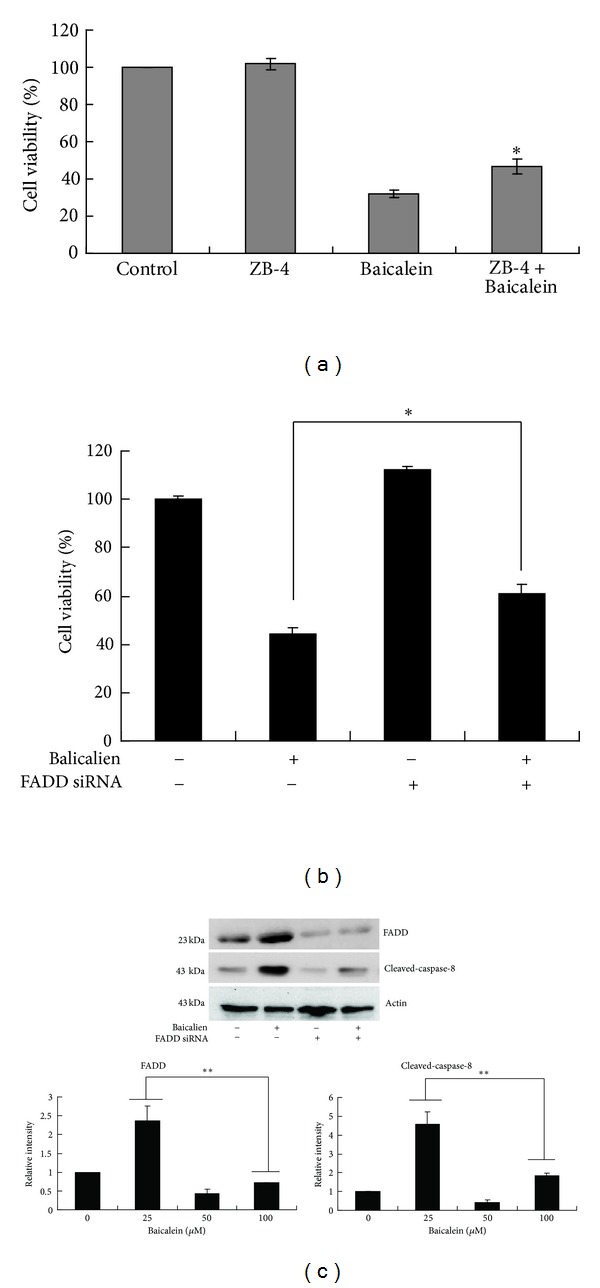
The effects of the Fas antagonistic antibody and transient transfection of FADD siRNA on cell viability in CCRF-CEM cells. (a) Cells pretreated with the Fas antagonistic antibody, ZB-4, were incubated with baicalein for 24 h, after which the cell viability was determined by the MTT assay. The data are presented as the mean ± SD in triplicate wells. Similar results were obtained in two independent experiments. *P < 0.05, compared with the baicalein only group. (b) Cells were transiently transfected with FADD siRNA, followed by baicalein exposure for 24 h. Then, the cells were examined for cell viability and (c) caspase 8 activation. Bands were analyzed by ImageJ and normalized to actin. The data in (b) are presented as the mean ± SD in triplicate wells. Similar results were obtained in two independent experiments. The data in (c) are representative of two independent experiments showing similar results. Means ± SD of the two independent experiments is presented with bar graph in the bottom. *P < 0.05, compared with the baicalein only group. **P < 0.01, compared with the baicalein only group.

We used the Fas neutralizing antibody ZB4 to examine whether the Fas/FasL interaction was necessary in baicalein-induced apoptosis. As shown in Figure 7(a), inhibition of the ligand-binding to Fas receptors could partially recover cell viability, indicating that the FasL/Fas interaction was necessary here to trigger apoptosis. Because trimerization of the Fas and TRAIL receptors leads to recruitment of the Fas-associated death domain (FADD), an adaptor molecule that recruits and activates caspase-8 [25], we further used FADD siRNA to confirm the association of the death receptor-caspase 8-tBid and baicalein-induced mitochondrial pathways. As illustrated in Figures 7(b) and 7(c), our results show that the silencing of FADD could partially recover the cell viability and repress caspase-8 activation. Overall, these results suggest that caspase-8 activation by baicalein is mostly dependent on death receptor signaling and that the death receptor-caspase 8-tBid pathway participates in, but is not the only trigger for, the mitochondria-mediated apoptosis induced by baicalein. 

### 3.8. Baicalein Triggers an Increase in the Intracellular Ca^2+^ Level

**Figure 8:**
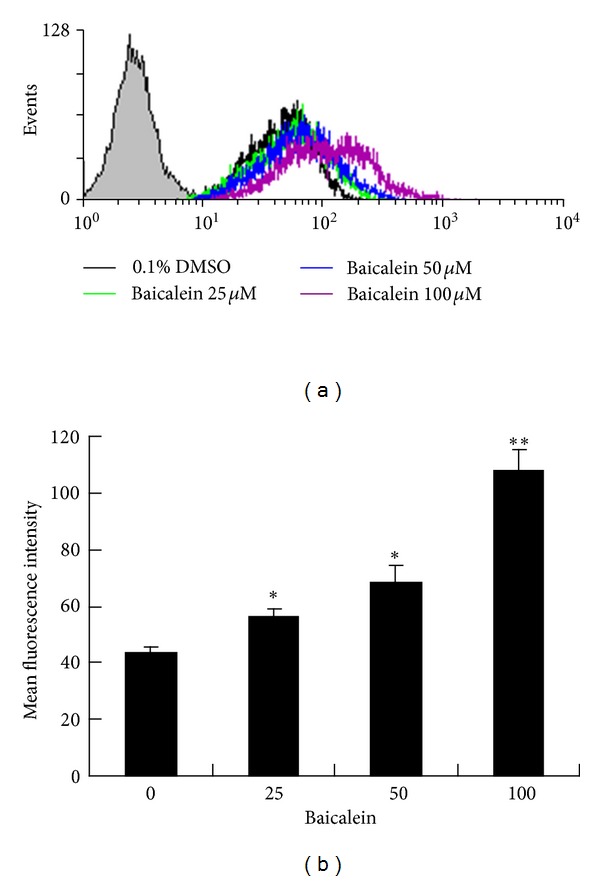
The effect of baicalein on the intracellular Ca^2+^ concentration. (a) CCRF-CEM cells were stained with Fluo-3 AM, followed by flow cytometry analysis 24 h post various concentrations of baicalein treatment. (b) The mean fluorescence intensities for the Ca^2+^ concentration were plotted. The data are represented as the mean ± SD in duplicate tests. Similar results were obtained in two independent experiments. *P < 0.05, compared with the 0.1% DMSO control group. **P < 0.01, compared with the 0.1% DMSO control group.

Intracellular free calcium ([Ca^2+^]_*i*_) has been known to enhance apoptosis at early and later stages of the apoptotic process [26]. In an attempt to determine whether Ca^2+^ plays a role in baicalein-induced apoptosis, the intracellular Ca^2+^ level is measured by a Ca^2+^-sensitive fluorescent dye, Fluo-3 AM, following 24 hr of baicalein exposure. As illustrated in Figures 8(a) and 8(b), baicalein caused a substantial increase in the intracellular Ca^2+^ at a higher dose (100 *μ*M) but seemed to cause only slight increases at lower doses (≦50 *μ*M). However, many apoptotic events were already quite obvious at 50 *μ*M of baicalein treatment. This implies that Ca^2+^ may enhance apoptosis at higher doses (≧100 *μ*M) of baicalein but seems to play less important roles in apoptosis at lower baicalein doses (≦50 *μ*M).

### 3.9. Combination of Baicalein and Vincristine Results in an Enhancement in the Therapeutic Efficacy

**Figure 9:**
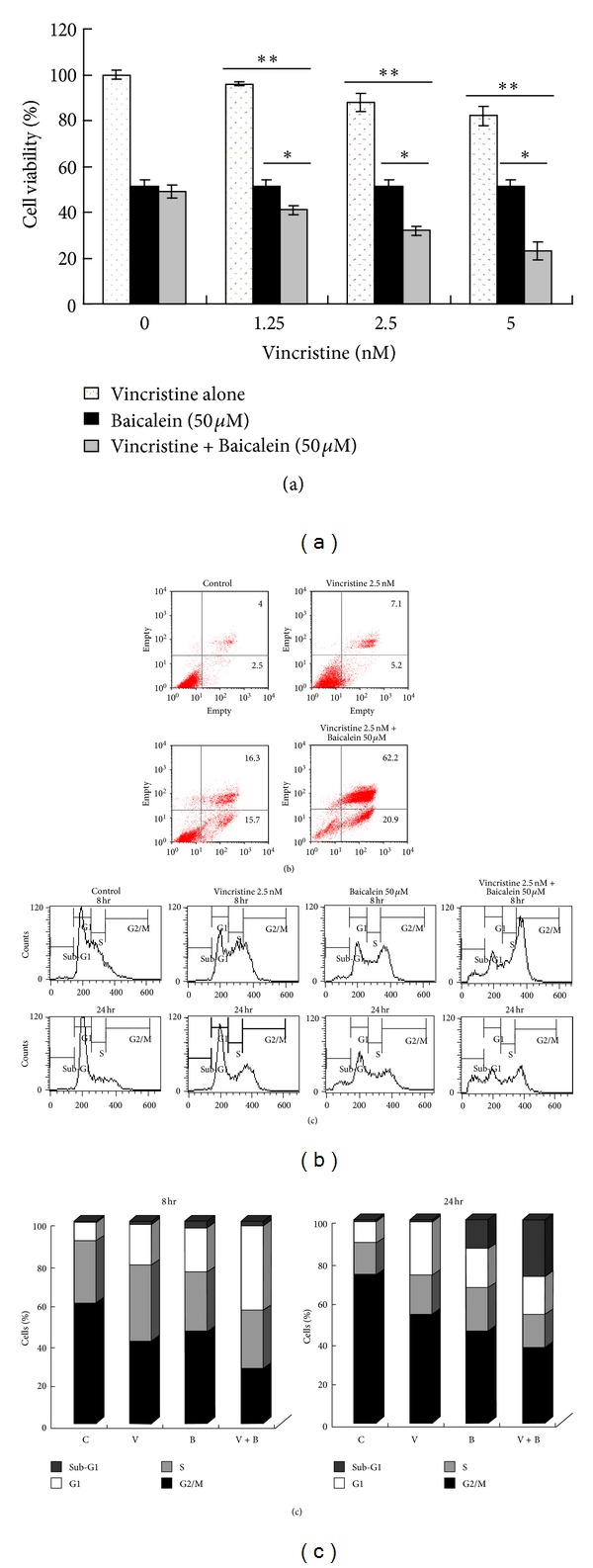
The combined therapeutic efficacy of baicalein and vincristine in CCRF-CEM cells. (a) 4 × 10^4^ cells/well were seeded onto 24-well culture plates, followed by incubation with the indicated doses of baicalein, vincristine or both for 24 h. The cell viability was then determined by the MTT assay. The data are presented as the mean ± SD from triplicate wells. Similar results were obtained in three independent experiments. **P < 0.01, compared with the group treated with vincristine alone. *P < 0.05, compared with the group treated with baicalein alone. (b) Cells were seeded at 3 × 10^5^ cells/dish onto 6-cm culture dishes and incubated with the indicated doses of baicalein and/or vincristine for 24 h. After staining with annexin V-FITC and propidium iodide (PI), the cells were analyzed by flow cytometry. The data are representative of two independent experiments showing similar results. (c) Cells were treated with the indicated concentrations of baicalein for 8 or 24 h, after which the cell cycle analysis was determined by propidium iodide (PI) staining and subsequent flow cytometry analysis. The data are representative of two independent experiments showing similar results.

Baicalein is toxic to cancer cells but displays low cytotoxicity for human normal cells [10]. Because vincristine can cause neurological toxicities when used in high doses, the combined therapeutic effect of baicalein with moderate doses of vincristine is worthy of investigation. Because a previous study had shown that the IC50 of vincristine in CCRF-CEM cells was approximately 9 nM with 24 h of incubation [27], lower doses (1.25, 2.5, and 5 nM) of vincristine were combined with baicalein to evaluate the therapeutic efficacy. As shown in Figure 9(a), our cell viability assay results demonstrate that baicalein clearly enhances the antileukemic effect of vincristine and the combined efficacy shows synergistic effect. Additionally, the enhancing effect is most obvious when baicalein is combined with moderate doses of vincristine (2.5~5 nM). In addition, an interaction (*P* < 0.01) is found between baicalein and vincristine based on a two-way ANOVA with Bonferroni's test. Flow cytometry using annexin V/PI staining also confirmed the enhancement in therapeutic efficacy when baicalein and vincristine were used in combination (Figure 9(b)). Furthermore, we preceded the cell cycle analysis in the settings of drug-combinations at 8 h and 24 h. As shown in Figure 9(c), the results validate that 50 *μ*M of baicalein shows M-phase blocking effect at 8 h, and a great portion of G2-M phase-arrested cells have died at 24 h, resulting in a decrease in G2-M phase percentage and an increase in sub-G1 phase percentage at 24 h. With respect to drug-combinations, the percentage of G2-M phase under combined treatment is much greater than baicalein or vincristine monotherapy at 8 h, and the sub-G1 percentage under combined treatment is greater than the sum of that under each monotherapy at 24 h. In addition, we have determined the combination effect on cell viability of baicalein and vincristine according to “Drug Synergism: Its Detection and Applications” reported by Tallarida [13], the resulting isobologram indicates that the combination effect of baicalein and vincristine is synergistic, and the synergistic effect is most obvious when baicalein is combined with moderate doses of vincristine (2.5~5 nM). The combination effect of 50 *μ*M baicalein and 1.25 nM vincristine is more likely to be additive.

## 4. Discussion

In the present study, we described the cellular and molecular events underlying baicalein-induced apoptosis in childhood acute lymphoblastic leukemia CCRF-CEM cells. Mitochondrial membrane permeabilization and cytochrome c release into the cytosol are early events in apoptotic signaling because the intrinsic and extrinsic pathways can converge at the mitochondrial level [14–17]. Antiapoptotic members of the IAP family also regulate the mitochondrial pathway [18]. In this study, our data reveal that the protein expression level of the anti-apoptotic XIAP and Survivin was reduced 24 h post baicalein treatment in CCRF-CEM cells (Figure 3(c)). Caspase-8 is known to propagate the apoptotic signal by direct cleavage and activation of downstream caspase-3 [19]. It is also well known that caspase-8 can trigger Bid cleavage into truncated Bid, which subsequently leads to the release of cytochrome c from the mitochondria, thereby triggering the activation of caspase-9 [19]. Membrane death receptor mediated apoptosis is known to be one of the pathways leading to caspase-8 activation [24]. Upon death receptors activation via ligation, the death receptors recruit the adapter molecule FADD to the death domain, which is also present on FADD, followed by activation of caspase-8 [25]. In this study, we observed silencing of the FADD attenuated cell death and caspase-8 activation (Figure 7(b)). In addition, we also observed an upregulation of DR4, DR5, FasL, and Fas (Figure 6). One previous study demonstrated that baicalein can induce DR5 protein expression in colon and prostate cancer cells [12]. Additional death receptors were found to be upregulated by baicalein in CCRF-CEM cells. These results suggest that the cascade signaling from the Fas/Fas ligand and DR4, DR5/TRAIL system to caspase 8-tBid plays a role in the apoptotic killing of CCRF-CEM cells by baicalein. 

Although Ca^2+^ is thought to be an enhancer of apoptosis, it may not play a prominent role under the efficacious dose (50 *μ*M) of baicalein that induce apoptosis in CCRF-CEM cells. This conclusion was drawn because many apoptotic events (mitochondrial membrane potential loss, cytochrome c release, caspase 9, 8, 3 activation, etc.) were already quite obvious at 50 *μ*M of baicalein treatment, but a substantial increase in intracellular Ca^2+^ appeared only after 100 *μ*M of baicalein treatment (Figure 8). Ca^2+^ may participate in, but does not seem to be the key player for, baicalein-induced apoptosis in CCRF-CEM cells under the efficacious dose of baicalein (50 *μ*M).

 In addition, 50 *μ*M of baicalein was chosen for most of our experiments, including the combination therapy, because the IC50 of baicalein in CCRF-CEM cells at 24 hr is approximately 48 *μ*M (Figure 1) and most of the apoptotic events are obvious at 50 *μ*M. In addition, one previous study on human promyelocytic leukemia HL-60 cells also found that an apparent reduction in cell viability was observed only after 24 h of 50 *μ*M of baicalein treatment [28].

Baicalein is known to be a potent anti-oxidant for incoming oxidative stress. There is evidence of this from several studies; baicalein exhibited a neuroprotective effect on hydrogen peroxide-mediated oxidative stress in PC12 cells [29] and on TG- or BFA-induced ROS accumulation in hippocampal neuronal cells [30]. Baicalein is also a potent antioxidant against cardiomyocytes ischemia/reperfusion injury [31]. Although increased ROS generation has been reported in several previous studies, there may be different effects in other cell types. This hypothesis was verified in a previous study in which 12 h after treatment with 40 *μ*M of baicalein, the intracellular ROS level rose moderately in PC3 prostate cancer cells, but the ROS level did not increase in SW480 colon cancer cells [12]. Another recent study on colon cancer cells reported findings that are similar to our results; the authors found that baicalein inhibits colon cancer cell proliferation and reduces reactive oxygen species (ROS) [11]. They also found that baicalein treatment has no effect on normal epithelial cells [11]. In support of this finding, two other studies found that baicalein hardly induces apoptosis in normal human cells, such as blood cells and hepatocytes [10, 12]. This phenomenon could be explained by a recently accepted concept that antioxidants exhibiting antiproliferative effects can be lethal in mitotically active cells, such as cancer cells but are not toxic to slow-growing non-cancer cells [32–34]. Two important messages are delivered here. Slow-dividing cells can easily escape from baicalein-induced apoptosis and are even protected by the antioxidant effect of baicalein when facing oxidative stress. However, baicalein tends to act more selectively on fast-growing cancer cells, rendering it an effective drug for cancer treatment. Its neuroprotective effects further increase its benefits when combined with vincristine, which has neurotoxic side effects at higher doses. 

In addition, this is the first report demonstrating the apoptosis-enhancing effect of baicalein when used in combination with vincristine. This enhancement in therapeutic efficacy is possibly attributed to the fact that both drugs are M-phase blockers. Vincristine inhibits cell division during early mitosis by binding to the tubulin monomers and preventing spindle microtubule formation. A variety of human cancer cell lines express survivin proteins, which are located on the mitotic phases and regulate mitotic progression. Survivin is an inhibitor of apoptosis that is expressed in various human cancer cells but is undetectable in most normal adult cells [35, 36]. Survivin has functions in both antiapoptosis and the promotion of mitosis in cancer cells [36, 37]. The stability of survivin is a result of the protein phosphorylation at Thr^34^ by the mitotic kinase complex CDC2/cyclin B1 [38, 39]. In our studies, baicalein reduced survivin protein expression (Figure 3(c)) and increased G_2_-M phase arrest at 8 h (Figure 9(c)) in CCRF-CEM cells. In addition, the percentage of G2-M phase under combined treatment was more than baicalein or vincristine monotherapy at 8 h, and the sub-G1 percentage under combined treatment was greater than the sum of that under each monotherapy at 24 h. Although the detailed mechanism for the enhancement in therapeutic efficacy in combination therapy is unclear, this phenomenon might be explained by that the simultaneous mitotic blocking stress exerted on CCRF-CEM cells by baicalein and vincristine tends to advance the M phase-arrested cells to enter apoptosis earlier. Our data indicate that the combination of baicalein and vincristine results in a synergistic therapeutic efficacy. More importantly, the use of baicalein in combination therapy not only lowers the required dose of vincristine, thus decreasing the related peripheral neuropathy, but baicalein may also alleviate some common chemotherapeutic side effects, such as nausea, vomiting, diarrhea, and skin rashes. This result was anticipated because baicalein is also used in traditional Chinese medicine to treat nausea, vomiting, diarrhea, stomach discomfort, and allergic and inflammatory conditions, such as arthritis, dermatitis, bronchitis, and asthma.

However, the combined cytotoxic effect of baicalein with methotrexate (MTX) was not as good as with vincristine (Supplementary Material available online at http://dx.doi.org/10.1155/2013/124747). MTX is a folate analogue designed to inhibit dihydrofolate reductase. Reduced folate (tetrahydrofolate) is involved in the de novo synthetic pathways for the purine and pyrimidine precursors of DNA and RNA, which are required for cell proliferation; thereby, MTX has been used extensively to treat neoplastic diseases. Unlike baicalein, which is an M-phase blocker, MTX is an S-phase inhibitor. Because the S phase was not altered by baicalein in CCRF-CEM cells, the dispersed cellular stress was less likely to advance the M phase- and S phase-arrested cells to enter apoptosis earlier. Therefore, this combination is less likely to produce an enhanced cytotoxic effect. Despite the lack of enhanced cytotoxic effect when combined with MTX, baicalein still possesses many beneficial effects, such as the alleviation of some common chemotherapeutic side effects (nausea, vomiting, diarrhea, and skin rashes).

The enhanced therapeutic effect of baicalein used in combination with vincristine and the chemotherapeutic side effect-alleviating action of baicalein greatly enhance the potential value of incorporating baicalein into future treatment regimens for childhood ALL. Baicalein can be introduced during the induction, intensification and maintenance phases of childhood ALL treatment, and its anti-inflammatory action may also reduce the hepatotoxicity often observed during the maintenance phase of ALL treatment. 

In conclusion, baicalein is a potential alternative drug for treating childhood acute lymphocytic (lymphoblastic) leukemia. Our results also support the use of combining baicalein and vincristine to treat childhood ALL. However, we also face a problem. Although to our knowledge, baicalein is relatively safe and the optimum dose of baicalein has not been established, we admit that 50 *μ*M of baicalein may not be achievable under dietary conditions. Baicalein is easily absorbed from the gastrointestinal tract of rats and undergoes conversion to baicalein via a first pass glucuronidation process in the liver [40, 41]. Baicalin is a glucuronated form of baicalein, both of which have been shown to promote anti-angiogenic effects *in vitro* [42], inhibit the proliferation of prostate cancer cells, and induce cell death via apoptosis [43, 44]. Although 50 *μ*M of baicalein might not be achievable under dietary conditions, this concentration is achievable via intravenous administration. It will be more suitable for baicalein to be administered intravenously in combination chemotherapy. In fact, several baicalein studies have been conducted by iv injection at doses of 10~20 mg/kg [45, 46] which corresponds to about 60~120 *μ*M. A recent study by Tsai et al. has reported much improved stability of baicalein by intravenous injection after incorporation into nanostructured lipid carriers (NLCs). The plasma level of baicalein in NLCs was much higher and the half-life much longer than those in the free control [47]. By virtue of NLC technology, the feasibility of the use of intravenous baicalein in combination chemotherapy in the future is substantially enhanced.

## Supplementary Material

The combined therapeutic efficacy of baicalein with another commonly used chemotherapeutic drug methotrexate in CCRF-CEM cells. (a) 4×104 cells/well were seeded onto 24-well culture plates, followed by incubation with the indicated doses of baicalein, methotrexate or both for 24 h. The cell viability was determined by the MTT assay. The data are presented as the mean ± SD from triplicate wells. Similar results were obtained in two independent experiments.
