# Supplementary figures and images for: Baicalein Triggers Mitochondria-Mediated Apoptosis and Enhances the Antileukemic Effect of Vincristine in Childhood Acute Lymphoblastic Leukemia CCRF-CEM Cells
Source: Evid Based Complement Alternat Med. 2013 Jan 28;2013:124747. doi: 10.1155/2013/124747 (PMC3580913; doi:10.1155/2013/124747)

**Supplemental 1**

**
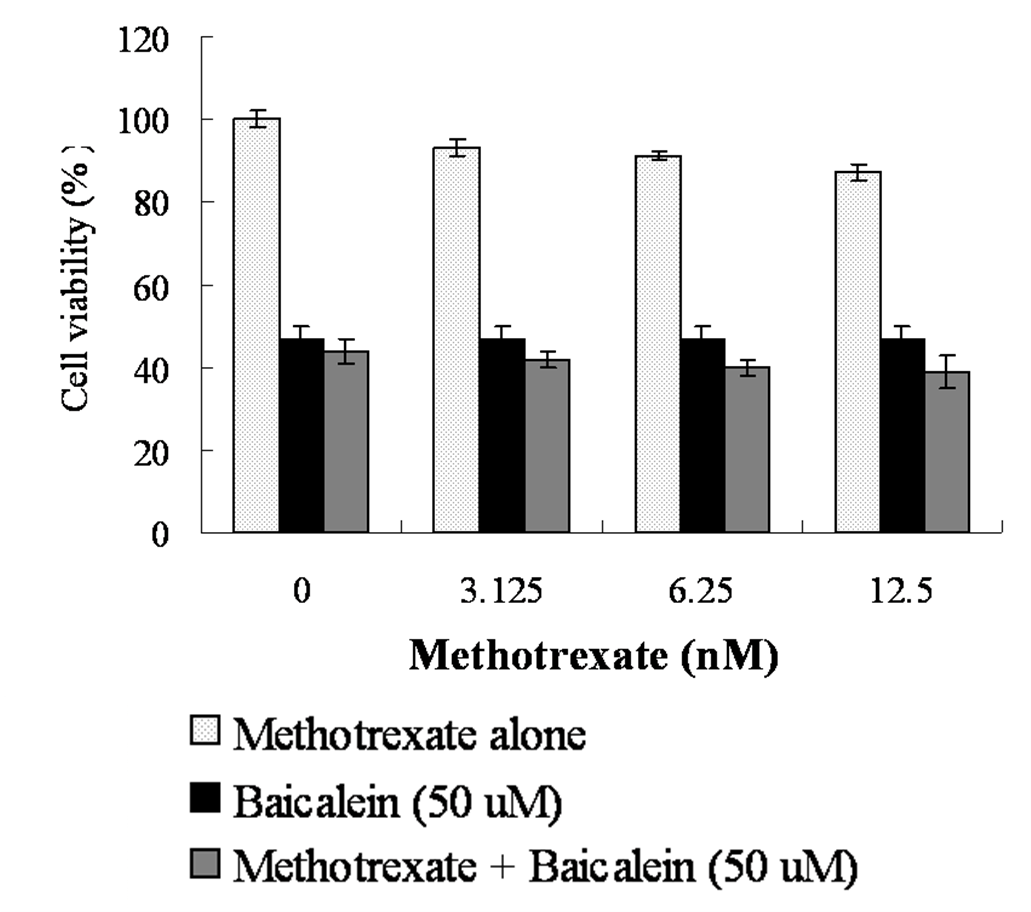
**

Supplement: Supplementary file 1 — The combined therapeutic efficacy of baicalein with another commonly used chemotherapeutic drug methotrexate in CCRF-CEM cells. (a) 4×10^4^ cells/well were seeded onto 24-well culture plates, followed by incubation with the indicated doses of baicalein, methotrexate or both for 24 h. The cell viability was determined by the MTT assay. The data are presented as the mean ± SD from triplicate wells. Similar results were obtained in two independent experiments. [file 124747.f1.doc]
